# Indicators of long-term sustainability of hand hygiene improvement and barriers in healthcare settings worldwide

**DOI:** 10.1186/1753-6561-5-S6-P262

**Published:** 2011-06-29

**Authors:** B Allegranzi, S Bagheri Nejad, D Pittet

**Affiliations:** 1 Patient Safety , World Health Organization, Geneva, Switzerland; 2 University of Geneva Hospitals, Geneva, Switzerland

## Introduction / objectives

Implementation of the WHO multimodal hand hygiene (HH) improvement strategy was successfully tested in 8 pilot sites in 2006-2008 with a significant improvement in HH compliance and other indicators. Indicators of long-term sustainability and barriers were evaluated 2 years later.

## Methods

Semi-structured telephone interviews with site coordinators were conducted in 2010 using a predefined set of questions to investigate the status of core activities of HH promotion and monitoring, barriers to improvement and indicators of long-term sustainability.

## Results

All coordinators accepted to be interviewed. The following indicators of long-term sustainability were identified: extension of HH promotion hospital-wide in sites where only a limited number of wards were initially involved in implementation; regular repetitionof HH training and preparation of new educational tools; poster refreshment and development of new reminders; continuation of HH compliance monitoring at least annually in 7/8 sites; national scale-up in 5/6 countries with no previous HH campaign. Barriers to long-term sustainability, especially in low-resource settings, were: resistance to behavioural change in some professional categories; high staff turnover and workload; understaffing; lack of dedicated human resources; discontinuation of support by leaders and of regular budget allocation, especially for alcohol-based handrub procurement.

## Conclusion

Long-term sustainability of the HH strategy was demonstrated by the continuation and reinforcement of core elements over time. Some identified barriers raise concerns and indicate the need for further improvement and some substantial changes, particularly in countries with limited resources.

## Disclosure of interest

None declared.

